# Prevalence of and prognosis for poor immunological recovery by virally suppressed and aged HIV-infected patients

**DOI:** 10.3389/fmed.2023.1259871

**Published:** 2023-10-19

**Authors:** Lina Fan, Penghui Li, Aiping Yu, Dan Liu, Ziyu Wang, Yue Wu, Defa Zhang, Meiyin Zou, Ping Ma

**Affiliations:** ^1^Department of Infectious Diseases, Tianjin Second People’s Hospital, Tianjin, China; ^2^Department of Surgery, Tianjin Second People's Hospital, Tianjin, China; ^3^Out-patient Department, Tianjin Second People's Hospital, Tianjin, China; ^4^Affiliated Infectious Disease Hospital of Nantong University, Nantong, Jiangsu, China; ^5^Tianjin Association of STD/AIDS Prevention and Control, Tianjin, China

**Keywords:** HIV/AIDS, inadequate immunological responders, non-AIDS related, cardiovascular disease, tumor

## Abstract

**Background:**

Antiretroviral therapy (ART) prolongs lifespan and decreases mortality of HIV infected patients. However, many patients do not achieve optimal immune reconstitution. The influence of non-optimal immune recovery on non-AIDS related diseases is not well defined in aged HIV-infected patients receiving ART.

**Methods:**

A retrospective study was conducted at Tianjin Second People’s Hospital, China to evaluate the association of an inadequate immunological response and non-AIDS diseases in HIV infected patients ≥60 years of age and virally suppressed for at least 2 years by ART.

**Results:**

The study included patients (*n* = 666) who initiated ART between August 2009 and December 2020. The prevalence of patients with an inadequate immunological response was 29.6%. The percentage of non-AIDS diseases such as hypertension, cardiovascular disease (CVD), diabetes, tumor, and chronic kidney disease (CKD) was 32.9, 9.9, 31, 4.1, and 13%, respectively. In addition to baseline CD4+ T cell counts, CVD and tumor were associated with poor immune reconstitution in aged Chinese HIV-1 infected patients. The adjusted odds ratios (95% confidence interval) were AOR 2.45 (95% CI: 1.22–4.93) and 3.06 (95% CI: 1.09–8.56, *p* = 0.03). Inadequate immunological response was associated with greater mortality (AOR: 2.83, 95% CI: 1.42–5.67, *p* = 0.003) in this cohort.

**Conclusion:**

These results tend to demonstrate appropriate drug selection at ART initiation and prevention of non-AIDS complications during ART decreased mortality of and an inadequate immunological response in aged HIV infected patients.

## Introduction

Antiretroviral therapy (ART) effectively controls HIV replication, increasing CD4+ T counts, and decreasing mortality. However, 9–40% of patients living with HIV (PLWH) fail to achieve CD4+ T cell count normalization and are referred to as “inadequate immunological responders,” (INRs) (1–3). Although the definition of INR was different in previous studies, the total CD4 T cell counts threshold of <200 or < 350 cells/μl at 2 years after ART initiation and persist HIV viral load undetectable or less than 20 copies/ml was more popular. Previous studies found that INRs are at greater risk of morbidity, mortality (4, 5), and non-AIDS related events.

ART has improved patient lifespan and changed HIV from a life-threatening illness to a chronic one. As a result, the number of aged HIV infected patients has increased. Aged HIV infected patients receiving ART have a reduced immune response (6, 7). At same time, aging with HIV infection has significant challenges, including inflammation due to chronic immune activation, cardiovascular disease, bone loss, and non-AIDS related cancers (8). According the literature, INR was associated with increased risk of non-AIDS defining events such as cardiovascular disease and even death.

The persons aged 65 years and over was defined as senior citizen (9)^.^ However, UNAIDS records PLWH aged 50 or over need provide appropriate prevention and demand age-appropriate health services (10). Previous study indicated that aged 60 years and over was associate with poor immune recovery and higher mortality (11). But no previous study has investigated the prevalence and prognostic of INR and non-AIDS related diseases in this special population. This study identifies INR risk factors and the burden of non-AIDS diseases in aged PLWH. The relationship of INRs to non-AIDS diseases is also described.

## Methods

### Study population

This study was conducted at Tianjin Second People’s Hospital, China, which has been a hospital designated for the treatment of HIV since July 2004. This study’s methodology conformed to the Declaration of Helsinki and was approved by the ethics committee of Tianjin Second People’s Hospital. ≥ 60 years HIV positive patients receiving ART for more than 2 years, with suppressed virology, were included in this study. After authorization by the ethics committee, available patient data were obtained from the electronic medical records of the hospital.

**Figure 1:**
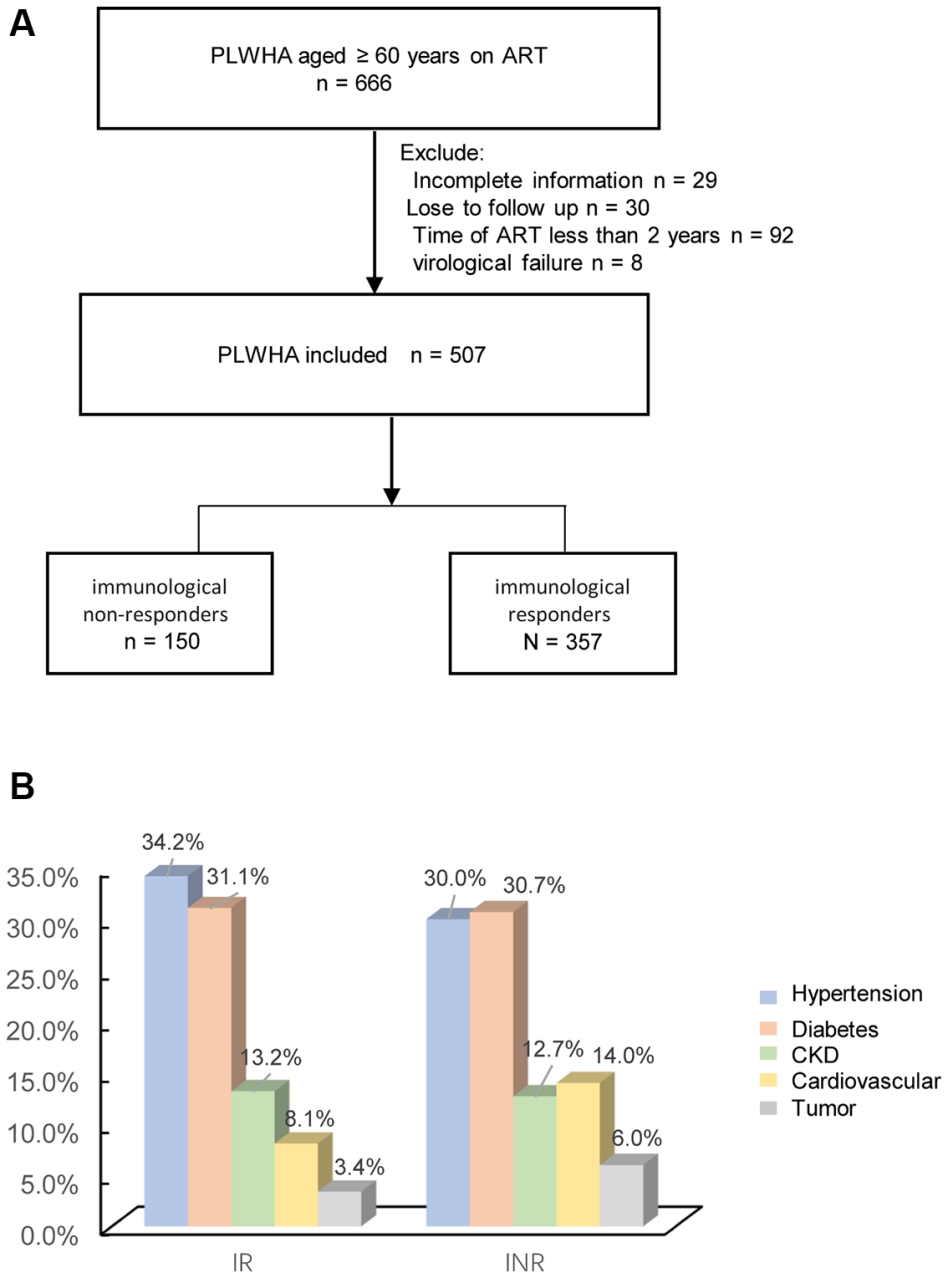
**(A)** Flow chart showing patient characteristics and selection; **(B)** the percentage of non-AIDS diseases.

Information was collected for approximately 666 ART patients ≥60 years of age, with virologic success through January 2023. Exclusion criteria included; incomplete information (29), loss to follow-up (30), ART time less than 2 years (92), and virologic failure (8). Data for 507 PLWH comprised the final study group. Of the 507 individuals, 150 patients had CD4 + T cell counts less than 350 cells/ml. A flow chart of patient characteristics and selection is presented in Figure 1.

### Definitions

CVD (myocardial infarction, stroke, coronary artery intervention, or death from chronic atherosclerotic cardiovascular disease); chronic renal disease, CKD (end-stage renal disease or eGFR <60 mL/min/ cm^2^); tumor (all cancers); diabetes (history of diabetes); hypertension (history of hypertension), INRs (CD4^+^T cell count below 350 cells/μl at 2 years after ART initiation, with undetectable plasma HIV RNA). Immunological responders (IRs, CD4^+^T cell counts above 350/μl at 2 years after ART initiation with with undetectable plasma HIV RNA). HIV virologic failure was defined as two consecutive measurements with HIV RNA > 200 copies/ml after 6 months of ART.

### Data collection

Retrospective data, including demographic and clinical/laboratory characteristics, were collected from medical records. Variables included; age, gender, HIV transmission route, co-infection with HBV, co-infection with HCV, diabetes, hypertension, CVD, tumor, and CKD. CD4^+^ T cell counts were stratified at baseline as >200, 50–199, and < 50 cells/μl.

### Statistical analysis

Statistical analysis was performed using SPSS 25.0 software (SPSS, Chicago, IL, United States) and GraphPad 8 (GraphPad Software, La Jolla, CA, United States). Because of skewed statistical distributions, numerical data are presented as medians with interquartile ranges (IQR); categorical variables are presented as percentages. Differences between two groups were analyzed using the Mann–Whitney U test (nonparametric). Logistic regression and Cox proportional regression models were used to evaluate risk factors associated with poor immune reconstitution after 2 years of ART, and mortality. Kaplan–Meier curves were computed for this cohort. Log-rank testing was carried out to evaluate differences in cumulative death in HIV-infected patients, with or without INR. Demographic variables (gender, age) and clinical/laboratory characteristics (CD4^+^ T cell counts at baseline, CVD, hypertension, diabetes, co-infection with HBV, and co-infection with HCV) were investigated as risk factors. A *p* value <0.05 was considered statistically significant.

## Results

### Demographic and clinical characteristics of HIV infected patients

A total of 507 aged PLWH were included in the analysis. Of these 150 (29.6%) had poor CD4 + T cell recovery (INR). The median age was 63 (IQR 61, 67), and the male to female ratio was 8.45:1. Twenty-three (4.5%) were co-infected with HBV, six (1.2%) were co-infected with HCV. The median BMI was 23.4 (IQR21.5, 25.6), the median CD4 T+ cell count at baseline was 188 (IQR72, 289) cells/μl. The percentages of non-AIDS associated diseases are shown in Figure 1B. The percentages of hypertension, CVD, diabetes, tumor, and CKD were; 32.9% (167), 9.9% (50), 31% (157), 4.1% (21), and 13% (66), respectively. In this cohort, 38/507 (7.6%) of the patients died with 3,547 person-years of follow-up. Patients with INR had lower CD4^+^T cell counts (65 vs. 246, *p* < 0.001), greater percentage of CVD (13.6% vs. 8.1%, *p* = 0.046), and a greater death ratio (13.1% vs. 4.2%). All descriptive statistics for study variables are presented in Table 1. The laboratory data after 2 years ART was shown in Supplementary Table S1.

**Table 1:** Comparison of characteristics between IR and INR in aged HIV infected patients.

Characteristics	Total *n* = 507	IR *n* = 357	INR *n* = 150	*p* vales
Age (Year)	63 (61, 67)	63 (61, 67)	64 (62, 68)	0.07
Male	448 (88.4%)	313 (87.7%)	135 (90%)	0.46
HBV co-infection	23 (4.5%)	18 (5%)	5 (3.3%)	0.40
HCV co-infection	6 (1.2%)	5 (1.4%)	1 (0.7%)	0.49
BMI (kg/m^2^)	23.4 (21.5, 25.6)	23.3 (21.6, 25.5)	23.0 (20.7, 25.2)	0.24
Duration of ART (years)	6.5 (4.4, 8.5)	6.6 (4.5, 8.6)	6.1 (4.3, 8.3)	0.003
Baseline Laboratory Data				
CD4 (cells/μl)	188 (72, 289)	246 (139, 326)	65 (22, 161)	<0.001
CD4/CD8 ratio	0.20 (0.10, 0.32)	0.25 (0.13, 0.36)	0.11 (0.04, 0.18)	<0.001
Glucose (mmol/L)	5.99 (5.4, 7.0)	6.0 (5.5, 7.0)	5.8 (5.3, 6.8)	<0.037
Cholesterol (mmol/L)	4.2 (3.7, 4.8)	4.2 (3.7, 4.8)	4.2 (3.6, 4.9)	0.938
Triglycerides (mmol/L)	1.4 (1.1, 1.8)	1.3 (1.1, 1.8)	1.5 (1.1, 1.9)	0.35
Non-AIDS combination *n* (%)				
Hypertension	167 (32.9)	122 (34.2)	45 (30)	0.36
Cardiovascular	50 (9.9)	29 (8.1)	21 (14)	0.04
Diabetes	157 (31)	111 (31.1)	46 (30.7)	0.74
Tumor	21 (4.1)	12 (3.4)	9 (6)	0.17
CKD	66 (13)	47 (13.2)	19 (12.7)	0.88
ART received at initiation *n* (%)				<0.01
NNRTI based	377 (74.4)	280 (78.4)	97 (64.7)	
PI based	62 (12.2)	44 (12.3)	18 (12)	
INSTI based	68 (13.4)	33 (9.2)	35 (23.3)	
Switching during ART *n* (%)	303 (60.5)	186 (60.0)	117 (61.3)	0.78
Death *n* (%)	38 (7.5)	16 (4.5)	22 (14.7)	<0.001

Means evaluation based on Q3 (Q1–Q4). ART, antiretroviral therapy; CKD, chronic kidney disease; NNRTI, non nucleotide reverse transcriptase inhibitors; PI, protease inhibitor; INSTI, integrase strand transfer inhibitor; BMI, body mass index.

### Cardiovascular diseases associated with higher risk of poor CD4+ T cell recovery

The multivariate logistic regression model found the following associations with poor immune reconstitution in elderly Chinese HIV-1 infected patients. These were; CVD (adjusted odds ratio (AOR): 2.45, 95% confidence interval (CI): 1.22–4.93, *p* = 0.01), tumor (AOR: 3.06, 95% confidence interval: 1.09–8.58, *p* = 0.03), baseline CD4^+^ T cell counts 50–199 cells/μl (AOR: 5.03, 95% confidence interval: 2.97–8.52, *p* < 0.001), and CD4^+^ T cells counts <50 cells/μl (AOR15.48, 95% CI:8.40–28.53, *p* < 0.001). Associations with decreased probability of poor CD4+ T cell recovery were protease inhibitor (PI) based (AOR: 0.32, 95% CI: 0.14–0.73, *p* = 0.01) and integrase strand transfer inhibitor (INSTI) based (AOR: 0.42, 95% CI: 0.23–0.76, *p* = 0.01), Table 2.

**Table 2:** Risk factors for poor immune reconstitution after 24 months of ART.

Variables	df	Univariate analysis OR (95%CI)	*p* value	Multivariate analysis OR (95%CI)	*p* value
CD4 counts at baseline					
>200	1				
50–199		5.04 (2.94, 8.65)	<0.001	5.03 (2.97, 8.52)	<0.001
<50		16.86 (9.00, 31.62)	<0.001	15.48 (8.40, 28.53)	<0.001
ART at initiation	2				
NNRTI based		1		1	
PI based		0.29 (0.12, 0.68)	0.004	0.32 (0.14, 0.73)	0.007
INSTI based		0.39 (0.21, 0.72)	0.003	0.42 (0.23, 0.76)	0.004
Cardiovascular diseases	1				
No		1		1	
Yes		2.66 (1.29, 5.51)	0.01	2.45 (1.22, 4.93)	0.01
Tumor	1				
No		1		1	
Yes		2.78 (0.98, 7.83)	0.06	3.06 (1.09, 8.56)	0.03
Switching drugs					
No	1	1			
Yes		0.78 (0.48, 1.24)	0.28		
Hypertension					
NO		1			
Yes		0.92 (0.57, 1.49)	0.73		
Diabetes	1				
No		1			
Yes		0.94 (0.58, 1.58)	0.83		
CKD	1				
No		1			
Yes		0.55 (0.25, 1.21)	0.14		
Gender					
Male		1	–		
Female		1.76 (0.87, 3.58)	0.12		
HCV co-infection	1				
No		1	–		
Yes		2.11 (0.19, 23.49)	0.54		
HBV co-infection	1				
No		1			
Yes		1.54 (0.47, 5.05)	0.43		
Age	1	1.03 (0.99, 1.08)	0.13		
Duration of ART	1	1.00 (0.99, 1.08)	0.66		

ART, antiretroviral therapy; NNRTI, non nucleotide reverse transcriptase inhibitors; PI, protease inhibitor; INSTI, Integrase strand transfer inhibitor; df, degree of freedom.

### Outcomes for virologic suppressed PLWH after 2 years of ART

Thirty-eight (7.6%) patients died during 3,547 person-years of follow-up. The main causes of death were; tumor (12) (endometrial carcinomas *n* = 1, anal cancer *n* = 2, lung cancer *n* = 2, stomach cancer *n* = 2, pancreatic cancer *n* = 1, leukemia *n* = 1, cholangiocarcinoma *n* = 1, and unidentified cancer *n* = 2), CVD (*n* = 11), opportunistic infections (OIs) (*n* = 7) (*Mycobacterium tuberculosis n* = 1, pneumocystis pneumonia *n* = 3, co-infected with bacteria *n* = 2, co-infected with other viruses, Covid-19, *n* = 2). Among these, OIs were only found in the INR group. Log-rank testing showed a significant difference between patients with and without CVD (*p* < 0.001), INR (*p* < 0.001), and tumor (*p* < 0.001).

The multivariate Cox logistic regression model found the following associated with increased mortality in elderly Chinese HIV-1 infected patients after 2 years of ART, Table 2. These were; HIV infection with CVD (adjusted odds ratio (AOR): 5.60, 95% CI: 2.41–13.04, *p* = 0.001), tumor (AOR: 7.09, 95% CI: 2.97–16.93, *p* < 0.01), INR (AOR: 2.83, 95% CI: 1.42–5.67, *p* = 0.003), and older age (AOR: 1.09, 95% CI: 1.04–1.15, *p* = 0.01). Change in ART drugs during ART (AOR: 0.14, 95%CI 0.06–0.37) was associated with decreased mortality (Table 3).

**Table 3:** Cox regression model for deaths in aged HIV infected patients.

Variables	df	Univariate analysis OR (95%CI)	*p* value	Multivariate analysis OR (95%CI)	*p* value
Age	1	1.11 (1.03, 1.15)	0.002	1.09 (1.04, 1.15)	0.01
Tumor	1				
No		1		1	
Yes		6.92 (2.80, 17.10)	<0.001	7.09 (2.97, 16.93)	<0.001
Diabetes	1				
No		1			
Yes		0.34 (0.11, 0.97)	0.05	0.32 (0.11, 0.94)	0.04
Cardiovascular diseases	1				
No		1		1	
Yes		4.74 (1.89, 11.85)	0.001	5.60 (2.41, 13.04)	0.001
Immunological non response	1				
No		1		1	
Yes		2.89 (1.37, 6.08)	0.005	2.83 (1.42, 5.67)	0.003
Switching drugs	1				
No		1			
Yes		0.18 (0.07, 0.48)	0.01	0.14 (0.06, 0.37)	<0.001
ART at initiation	2				
PI based		1			
NNRTI based		1.65 (0.48, 5.61)	0.42		
INSTI based		2.91 (0.68, 12.40)	0.15		
CKD	1				
No		1			
Yes		0.40 (0.07, 2.47)	0.32		
Gender					
Male		1	–		
Female		1.52 (0.35, 6.58)	0.58		
HBV co-infection	1				
No		1			
Yes		0.94 (0.12, 7.43)	0.94		
HCV co-infection	1				
No		1	–		
Yes		10.96 (0.76, 158.73)	0.08		
Hypertension					
No		1			
Yes		0.70 (0.28, 1.77)	0.45		

ART, antiretroviral therapy; NNRTI, non nucleotide reverse transcriptase inhibitors; PI, protease inhibitor; INSTI, Integrase strand transfer inhibitor; df, degree of freedom.

## Discussion

In this study, 150 (29.6%) aged PLWH were INR, which is consistent with a previous study (12). Aged INRs with successful ART had a higher mortality and exhibited greater CVD and lower baseline CD4+ T cell counts. The increased mortality of patients with INR was associated with higher OIs, which demonstrates the importance of OIs in aged INR patients.

Although ART has reduced worldwide HIV related mortality and morbidity, INR patients with virologic suppression still have a greater probability of poor outcomes and no obvious CD4+ T cell recovery. Baseline CD4+ T cell counts, advanced age, co-infection with HCV, thymic dysfunction, immune activation, and genetic factors contribute to poor immune recovery (13, 14). By statistical analysis, baseline CD4+ T cell counts was associated with INR. We also found that non-AIDS diseases and initial ART drug combinations were associated with INR. These results demonstrate the importance of ART third drug selection as well as the prevention of non-AIDS diseases in PLWH, as the means by which to decrease the probability of INR.

Persistent inflammation and immune activation exacerbate tissue damage in PLWH, increasing the risk for non-AIDS-related co-morbidities that are commonly observed in PLWH and advanced age (15). Further, long term ART and advanced age are associated with CVDs such as heart diseases and stroke. In this study’s cohort, the prevalence of CVD was lower than in HIV/AIDS patients more than 60 years since the IPEC/FIOCRUZ cohort (15) (9.9% vs. 27.5%), but comparable with the rate of a Spanish HIV/AIDS cohort (13%) (16). CVD self-report without health care personnel examination may explain the lower rate found in this study.

Hypertension was found in 33.1% of all patients in this study, which is greater (33.1% vs. 30.3%) than rates reported for the IPEC/FIOCRUZ cohort. In this study, there was a greater prevalence of diabetes among elderly patients (31% vs. 4.6%) compared to the IPEC/FIOCRUZ cohort, which was also greater than rates observed for the Chinese general population (≥ 60 years, 31% vs. 26.7) (17). Genetic differences and dietary habits may explain these results.

**Figure 2:**
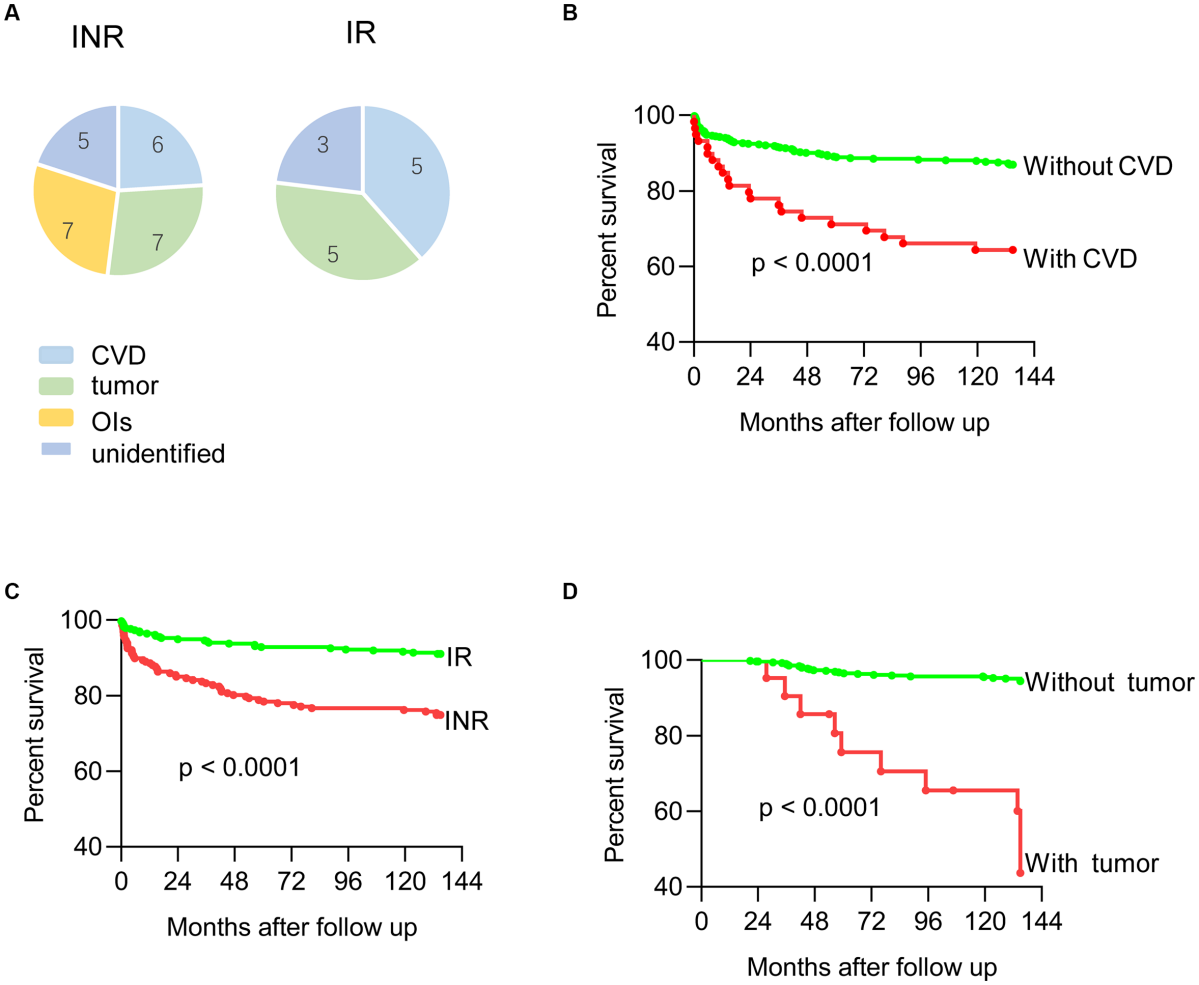
**(A)** Death of immune non-responders and immune responders; **(B)** mortality curves of HIV infected patients with or without CVD; **(C)** mortality curves of HIV infected patients with or without tumor; **(D)** mortality curves of HIV infected patients with or without immune recovery.

In a previous study the main risks for death in patients receiving ART have changed from OIS to non-AIDS-defining malignancies and CVD (18, 19). For this cohort of patients, seven deaths (18%) were due to OIs, all of which were with in INR group. These findings suggest that aged HIV infected patients with poor immune recovery require careful follow-up consideration of CD4+ T cells counts, as a means by which to minimize mortality (Figure 2).

Previous studies found that death risk for patients receiving continuous ART with CD4+ T cell counts >500 cells/μl was similar to patients with full immune recovery. INRs have twice the number fatal and non-fatal non-AIDS diseases compared to IRs (20, 21). In this study, a greater mortality rate was found for INR patients, indicating that close clinical attention is required for INR patients, particularly those with OIs and non-AIDS diseases.

Treatment option at ART initiation for INR patients are still debated. INSTI-based was confirmed a faster viral load suppression and immune recovery than PI-based regimens after ART in early HIV infection (22). Another multicenter study observed INSTI-based initiation gained faster immune restoration but lose the priority in long time follow up (23). Intensified INSTI-based three drugs versus four drugs regimen include boost PI, showed comparable responses in immunological recovery after ART initiation (24). In this study, we also recommend the use of INSTI-based and PI-based, but not NNRTI- ART for treatment initiation.

There are limitations to this study. First, the inherent bias of a retrospectively designed study is a limitation. Second, the initiation and diagnosis of non-AIDS related diseases was not adequately recorded in the patients’ medical records. Third, data were only derived from the Tianjin HIV tertiary health center. Future studies should examine the effects of non-AIDS diseases on immune recovery of patients similar to those investigated by this study.

## Conclusion

In conclusion, this study identifies the risk factors for poor immune recovery of aged HIV infected patients. Baseline CD4+ T cell counts, CVD, tumor, and initial ART drug are strongly associated with poor immune recovery. Greater mortality and a greater risk for INR in an aged population argues for close monitoring of ART treatment effects and non-AIDS complication during ART. Switching ART drugs during treatment was associated with lower mortality rates, therefore, the initial treatment selection might affect mortality rates. Immune reconstitution differences after ART drug initiation demonstrate drug selection to be important for aged patients. Further research is needed to determine whether alterations in ART drug selection would improve immune reconstitution and better clinical outcomes.

## Data availability statement

The original contributions presented in the study are included in the article/Supplementary material, further inquiries can be directed to the corresponding authors.

## Ethics statement

The studies involving humans were approved by Medical Ethics committee of Tianjin Second People’s Hospital Approval certificate of Ethical Review [2020]49. The studies were conducted in accordance with the local legislation and institutional requirements. The participants provided their written informed consent to participate in this study.

## Author contributions

LF: Data curation, Formal analysis, Methodology, Writing – original draft. PL: Data curation, Validation, Formal analysis, Investigation, Writing – original draft. AY: Data curation, Investigation, Supervision, Writing – original draft. DL: Data curation, Methodology, Writing – original draft. ZW: Data curation, Writing – original draft. YW: Data curation, Writing – original draft. DZ: Data curation, Writing – original draft. MZ: Writing – original draft, Supervision, Validation. PM: Writing – original draft, Funding acquisition, Project administration.
