# Supplementary material for: Prevalence of and prognosis for poor immunological recovery by virally suppressed and aged HIV-infected patients
Source: Front Med (Lausanne). 2023 Oct 19;10:1259871. doi: 10.3389/fmed.2023.1259871 (PMC10625403; doi:10.3389/fmed.2023.1259871)
Supplement: Supplementary file 1 [file Table_1.docx]

| Characteristics | Total  n=507 | IR  n=357 | INR  n=150 | *P* vales |
| --- | --- | --- | --- | --- |
| CD4(cells/μl) | 486 (317, 693) | 585 (469, 726) | 237 (182, 295) | <0.001 |
| CD8 (cells/μl) | 822 (563, 1166) | 839 (587, 1172) | 786 (517, 1154) | 0.20 |
| Glucose (mmol/L) | 6.2 (5.6, 7.0) | 6.2 (5.7, 6.9) | 6.2 (5.6, 7.0) | 0.96 |
| Cholesterol (mmol/L) | 4.6 (4.0, 5.3) | 4.6 (3.9, 5.3) | 4.7 (4.1, 5.3) | 0.52 |
| Triglycerides (mmol/L) | 1.7 (1.2, 2.6) | 1.6 (1.2, 2.6) | 1.7 (1.2, 2.7) | 1.00 |

Supplementary Table 1 Comparison of laboratory data after 2-year ART between IRs and INRs in aged HIV infected patients

Note: means evaluation based on Q3(Q1-Q4).
